# Childhood Maltreatment and Life Satisfaction in Chinese Student Preschool Teachers: The Roles of Resilience and Social Support

**DOI:** 10.3390/bs12110438

**Published:** 2022-11-09

**Authors:** Xiaojing Liu, Shengkai Ji, Juan Jiang, Chen Chen

**Affiliations:** 1 Center for Educational Science and Technology, Beijing Normal University, Zhuhai 519087, China; 2 Pinghu Normal College, Jiaxing University, Jiaxing 314220, China; 3 Department of Preschool Education, Liaoning National Normal College, Shenyang 11032, China

**Keywords:** childhood maltreatment, life satisfaction, resilience, social support, student preschool teachers

## Abstract

Although some studies have explored the relationships between childhood maltreatment and life satisfaction, few studies have explored the pathways between those two variables in a sample of student preschool teachers. The current study, thus, attempts to explore the relationships between childhood maltreatment and life satisfaction in Chinese student preschool teachers and to examine the roles of resilience and social support in those relationships. A total of 1218 students majoring in early childhood education were recruited from three Chinese universities to attend this study. Self-reported questionnaires were used to collect data, and structural equation modeling was used to perform data analysis. Results showed that childhood maltreatment was negatively associated with life satisfaction in Chinese student preschool teachers; resilience and social support mediated those relationships. The findings suggest that childhood maltreatment not only has a direct relationship with life satisfaction, but also has an indirect relationship with life satisfaction via resilience and social support. Childhood maltreatment should be considered when enrolling student preschool teachers, and increasing levels of resilience and social support should be meaningful approaches when cultivating student preschool teachers who have experienced childhood maltreatment.

## 1. Introduction

Childhood experiences, especially childhood maltreatment, may impair the later development of emotions, behaviors, and brain structures and functions [1], and these severe outcomes of childhood maltreatment may be an important contributing factor to maladaptation. Life satisfaction, as a subjective perception of daily life events, has been considered as one index for well-being [2] which may be influenced by childhood maltreatment [3], while pathways between these two variables still need further exploration, particularly in Chinese samples.

Early childhood education (ECE) plays a significant role in individuals’ development, and various influential factors for enhancing the quality of ECE have been explored by extensive studies [4], such as studies on the characteristics of preschool teachers [5,6]. However, few studies have explored the effects of childhood maltreatment on preschool teachers’ later development.

Guided by ecological system theory, which posits that interactions between individuals and environments (e.g., the family context) have effects on individuals’ emotions, behaviors, and attributions, and mediating influencing factors influence those interactions [7], the current study attempts to explore the relationships between childhood maltreatment and life satisfaction and to examine some mediators in these relationships based on a sample of Chinese student preschool teachers.

### 1.1. Childhood Maltreatment and Life Satisfaction

Childhood maltreatment, including abuse and neglect, is an important worldwide public health issue due to its high prevalence and severe outcomes [8]. Chinese parents may be influenced by traditional Chinese culture, which may contribute to different interactional patterns with children than those performed by Western people. The prevalence of childhood maltreatment in the Chinese sample was 26.6% based on a meta-analysis, which might be higher than Western prevalence [9]. Moreover, childhood maltreatment impairs later development, as verified by a growing body of Chinese studies [10], which raises the importance of exploring childhood maltreatment in the Chinese cultural context.

Life satisfaction as an index of well-being can be influenced by childhood maltreatment. For example, a study found that childhood maltreatment was negatively associated with life satisfaction based on a cross-sectional study including 550 Korean young adults [11]. A Chinese study indicated that Chinese college students who have experienced childhood maltreatment had low levels of life satisfaction [12]. Similarly, some longitudinal studies confirmed these relationships. For instance, a study reported that childhood maltreatment predicted lower levels of life satisfaction than any other adverse childhood experiences based on a three-wave study with 6323 participants [13]. Although previous studies have verified that childhood maltreatment impairs later life satisfaction, few studies have explored those relationships in student preschool teachers.

### 1.2. Childhood Maltreatment, Social Support, and Life Satisfaction

Although the relationships between childhood maltreatment and life satisfaction have been explored by extensive studies, the pathways from childhood maltreatment to life satisfaction need further exploration. Social support is one of the coping strategies which may help individuals to reduce stress actions, become less nervous, and overcome other maladaptation. Some studies reported that social support might be impaired by childhood maltreatment. For example, a study indicated that physical abuse predicts low levels of social support based on 598 Americans [14]. These results were confirmed by a study based on 372 couples [15]. Moreover, social support is associated with life satisfaction. For instance, a study found that social support was positively associated with life satisfaction based on 362 Chinese college students [16]. A study reported that social support predicted high levels of life satisfaction in 275 Chinese young adults [17].

Similarly, some studies explored social support as a mediator in the relationships between childhood maltreatment and later development. For example, a study indicated that social support mediated the relationships between child abuse and social functioning among 152 Americans from low-income families [18]. A study indicated that social support mediated the relationships between childhood maltreatment and non-suicidal self-injury among 4799 Chinese college students [19]. Additionally, a study reported that social support mediated the relationships between child abuse and physical and mental health among 457 American adults based on a longitudinal study [20]. Specifically, child abuse impairs physical and mental health [21,22], and high levels of social support might influence the effects of child abuse on later development [23]. Although several studies have explored the bidirectional relationships between childhood maltreatment, social support, and life satisfaction, as well as the mediating effects of social support in the relationships between childhood maltreatment and later development, few studies have explored the roles of social support in the relationships between childhood maltreatment and life satisfaction, particularly in student preschool teachers.

### 1.3. Childhood Maltreatment, Resilience, and Life Satisfaction

Resilience, the ability to bounce back from adverse experiences, has been defined as a mental process of adaption when facing stress or trauma [24] which may be influenced by childhood maltreatment. For example, a study reported that individuals who have experienced childhood maltreatment present low levels of resilience [25]. Hong et al. (2018) indicated that childhood maltreatment was negatively associated with resilience among 268 American college students [26]. Similarly, a study reported that childhood maltreatment was negatively associated with resilience among 809 Chinese college students [27], and these results were confirmed by other studies [28].

Moreover, some studies have explored relationships between resilience and life satisfaction. For example, a study reported that resilience positively predicted life satisfaction based on a sample of 201 American college students [29]. A study reported that resilience was positively associated with life satisfaction among 282 Chinese college students [30]. A study indicated that resilience predicted later life satisfaction among 249 Chinese adolescents based on a longitudinal study [31], and these results were confirmed other studies [32,33].

Additionally, resilience has been explored as a mediator in the relationships between childhood maltreatment and later development. For example, a study reported that resilience mediated relationships between childhood maltreatment and mental health among 600 Chinese college students [34]. Specifically, individuals who have experienced childhood maltreatment may have increased risk of psychosis (e.g., affective and personality disorders; [35,36,37]), and high levels of resilience may help individuals to overcome the effects of childhood maltreatment on later development. A study reported that resilience mediated the relationships between childhood maltreatment and aggression among 809 Chinese college students [38]. Although resilience has been explored as a mediator between childhood maltreatment and later development, few studies have explored its role in the relationships between childhood maltreatment and life satisfaction, particularly in student preschool teachers.

### 1.4. The Current Study

Although a growing body of studies has explored the relationships between childhood maltreatment and life satisfaction, the pathways from childhood maltreatment to life satisfaction still need further exploration, particularly in the Chinese cultural context. China, as an ancient Eastern country, may have different cultures to Western countries, which may contribute to different interaction patterns between the child and the caregiver(s) than those performed by Western people. Traditional Chinese culture emphasizes internal attributions for daily life mistakes, which may influence the attribution ways of modern Chinese people. Moreover, Chinese culture is one of collectivism in which people construct selves based on their relations with others, which raises the importance of the roles of childhood experiences in later development. Student preschool teachers may have acquired much more knowledge about protecting children and positive parenting strategies which may help them to better understand childhood maltreatment and may cause them to have different views about childhood maltreatment than those people in other samples. Additionally, student preschool teachers who have experienced childhood maltreatment may use the same way that he or she was treated to treat the children in preschools, which highlights the necessity of exploring childhood maltreatment in student preschool teachers. This study, thus, attempts to examine the relationships between childhood maltreatment and life satisfaction and to explore the mediators in those relationships in a sample of Chinese student preschool teachers. We hypothesize that (1) childhood maltreatment impairs life satisfaction and (2) social support and resilience have mediating effects on the relationships between childhood maltreatment and life satisfaction. This study may broaden the study of preschool teacher education in the Chinese cultural context and raise the importance of the childhood experience of preschool teachers when exploring factors relating to quality of ECE as well as provide suggestions and evidence for selecting and training preschool teachers.

## 2. Method

### 2.1. Participants

The current study recruited participants from the Chinese Longitudinal Study of Preschool Teacher Students’ Development (CLSPTSD) research project. This project aims to explore student preschool teachers’ professional identity recognition, trajectory, and dynamic predictors in the Chinese cultural context. Based on this project, 1218 students majoring in ECE were recruited from three Chinese normal universities located in provinces of Zhejiang, Jiangsu, and Liaoning, China.

### 2.2. Data Collection Procedures

The current study followed four steps to collect data. First, three normal universities were randomly selected from three provinces based on economic development levels in China. Second, the corresponding researcher explained the aims and processes of the current study to cooperators in the three universities and received the final approvals. Third, 1218 student preschool teachers joined this study. Last, an on-line informed consent letter and the questionnaires were sent to the participants, and each participant received a small gift worth RMB 10 (USD 1.55) after completing the questionnaires. In addition, the present study was approved by the ethics committee of the researchers’ institutions, and all research procedures were safe for participants.

### 2.3. Measures

#### 2.3.1. Childhood Trauma Questionnaire Short Form (CTQ-SF)

The CTQ-SF is a 28-item, retrospective, self-reported questionnaire which is used to evaluate traumatic experiences in childhood, including various forms of abuse and neglect [39,40]. Due to its good psychometric properties, it has been translated into a variety of languages. The Chinese version of the CTQ–SF was translated and compiled by a Chinese group [41], and this adaption has been deemed valid and reliable among adolescents and young adults [42,43]. Five subscales that focus on assessing different aspects of child abuse and neglect are included in the Chinese version of the CTQ–SF: physical abuse (e.g., *was hit so hard by family*), sexual abuse (e.g., *was molested*), emotional abuse (e.g., *called stupid*, *lazy*, *or ugly by family*), physical neglect (e.g., *wear dirty clothes*), and emotional neglect (e.g., *felt loved*). Each subscale is composed of 5 items, and a minimization–denial subscale of 3 items is also included, and each item is rated by a 5-point Likert scale (1 = *never* to 5 = *always*), and high average scores indicate high levels of childhood maltreatment. The Chinese version subscales of physical neglect, emotional abuse, emotional neglect, and physical abuse were administered in the present study, and the Cronbach’s alpha of these four subscales and total scale were 0.64, 0.78, 0.89, 0.87, and 0.89, respectively.

#### 2.3.2. Life Satisfaction Scales Applicable to Chinese Adolescent Students (CASLSS)

The CASLSS is a 36-item, self-reported measure used to evaluate the satisfaction with life of Chinese adolescent students [44,45]. It has 6 dimensions, friendship (e.g., *My friends respect me*), family (e.g., *I like to stay with my parents*), study (e.g., *I have achieved an ideal academic achievements*), freedom (e.g., *No one has forced to do anything that I don’t like*), school (e.g., *Being at school makes me feel uneasy*), and environment (e.g., *I hope I can live in places other than where I am now*). Each item is rated by a 7-point Likert scale (1 = *completely inconsistent* to 7 = *completely consistent*), and high scores indicate high levels of life satisfaction. The CASLSS has acceptable reliability and validity in samples of Chinese college students [46,47]; the Cronbach’s alpha of the subscales of friendship, family, school, environment, freedom, study, and total scale in the current study were 0.83, 0.92, 0.86, 0.72, 0.85, 0.88, and 0.96, respectively.

#### 2.3.3. Connor-Davidson Resilience Scale (CD-RISC)

The CD-RISC is a self-reported rating instrument intended to assess resilience among adults [48]. The Chinese version of the CD-RISC, with 25 items, was developed by a group [49]. It has three subscales: tenacity (e.g., *Strong sense of purpose*), strength (e.g., *Able to adapt to change*), and optimism (e.g., *Can deal with whatever comes*). Each item is rated on a 5-point Likert scale (0 = *not at all true* to 4 = *true nearly all the time*), and high scores indicate high levels of resilience. The Cronbach’s alpha of the subscales of tenacity, strength, optimism, and total scale in the current study were 0.90, 0.92, 0.78, and 0.96, respectively. According to item parceling [50], the scale was broken into five parts, tenacity 1 (BT1), tenacity 2 (BT2), strength 1 (BS1), strength 2 (BS2), and optimistic (BO).

#### 2.3.4. Multidimensional Scale of Perceived Social Support (MSPSS)

The MSPSS is a 12-item, self-reported measure which aims to assess perceived social support (SP) [51]. Each item is rated by a 7-point Likert scale (1 = *very strongly disagree* to 7 = *very strongly agree*), and high scores indicate high levels of social support. A study reported that the MSPSS shows good reliability and validity in the Chinese cultural context [52]. The Cronbach’s alpha of this scale was 0.97 in the present study. According to item parceling [50], the scale was broken into four parts, social support 1 (FS1), social support 2 (FS2), social support 3 (FS3), and social support 4 (FS4).

### 2.4. Data Analysis

Before the overall data analysis, missing values and outliers were examined, and the questionnaires with missing data were also excluded. Descriptive analyses covered all variables of interest for the total sample. Pearson’s product–moment correlation analysis was conducted to examine the relationships between all variables via SPSS 21. All tests were two tailed for significance, and significance (*p*-value) was set at 0.05. Furthermore, structural equation models (SEMs) were performed via AMOS 22 to explore the relationships between childhood maltreatment and Chinese student preschool teachers’ life satisfaction. The detailed steps of the data analysis were as follows:

First, confirmatory factor analysis (CFA) was conducted to test the measurement models before testing the structural path models. Then, SEMs performed with the maximum likelihood method were used to estimate the model parameters. Second, goodness of fit was evaluated by using the following cut-offs for model fit indices: a root-mean-square error of estimation (RMSEA) value close to 0.08 [53], as well as a comparative fit index (CFI) and Tucker–Lewis index (TLI) value equal to or greater than 0.95 [53]. Third, bias-corrected bootstrap was conducted (1000 samples) to test the mediating effect [54]. Both direct and indirect effects of childhood maltreatment on life satisfaction were estimated, which generated a percentile based on confidence intervals (CI). In addition, age, gender, and family income were controlled in the SEMs as some other influential variables relating to life satisfaction.

## 3. Results

### 3.1. Descriptive Statistics

Results of the descriptive statistics and correlation analysis are presented in Table 1 and Table 2. Among the participants, 95.4% were girls (*n* = 1162), with an average age of 19.19 years (SD = 0.94). Furthermore, 48.7% of them (*n* = 605) were the only child in the family. In addition, participants’ monthly family income was diverse. Moreover, childhood maltreatment was negatively associated with Chinese student preschool teachers’ life satisfaction (*r* = −0.29, *p* < 0.01). Childhood maltreatment was negatively associated with resilience (*r* = −0.11, *p* < 0.01) and social support (*r* = −0.31, *p* < 0.01), which indicates that student preschool teachers who scored higher on childhood maltreatment had lower resilience and social support.

### 3.2. The Relationships between Childhood Maltreatment and Life Satisfaction

SEMs were used to measure the direct relationships between childhood maltreatment and life satisfaction in Chinese student preschool teachers. Results of the SEM indicated that childhood maltreatment was negatively associated with life satisfaction (β = −0.56, SE = 0.037, *p* < 0.001), and the structural model provided an acceptable fit to the data (RMSEA = 0.08, CFI = 0.956, TLI = 0.929); physical abuse was negatively associated with life satisfaction (β = −0.23, SE = 0.080, *p* < 0.001; RMSEA = 0.08, CFI = 0.967, TLI = 0.947); emotional abuse was negatively associated with life satisfaction (β = −0.41, SE = 0.044, *p* < 0.001; RMSEA = 0.05, CFI = 0.988, TLI = 0.976); physical neglect was negatively associated with life satisfaction (β = −0.43, SE = 0.033, *p* < 0.001; RMSEA = 0.07, CFI = 0.980, TLI = 0.958); and emotional neglect was negatively associated with life satisfaction (β = −0.48, SE = 0.026, *p* < 0.001; RMSEA = 0.08, CFI = 0.977, TLI = 0.952).

### 3.3. Resilience and Social Support Mediated the Relationships between Childhood Maltreatment and Life Satisfaction

**Figure 1:**
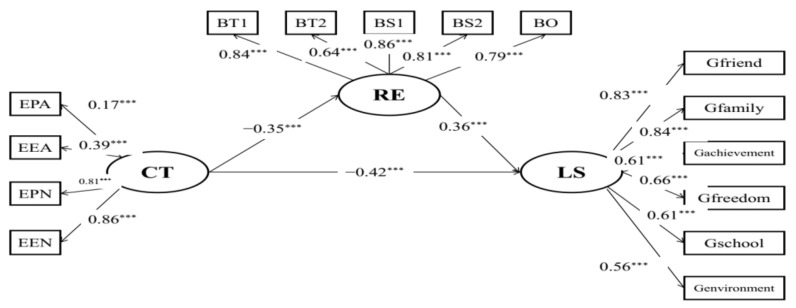
Mediation model of child maltreatment, life satisfaction, and resilience. EPA: physical abuse; EEA: emotional abuse; EPN: physical neglect; EEN: emotional neglect; BT1: tenacity 1; BT2: tenacity 2; BS1: strength 1; BS2: strength 2; BO: optimism; CT: childhood maltreatment; LS: life satisfaction; RE: resilience; *** *p* < 0.001.

Structural model A (Figure 1) with acceptable model fits (RMSEA = 0.078, CFI = 0.934, TLI = 0.915) reflected the mediating effects of resilience on the relationships between childhood maltreatment and life satisfaction. Childhood maltreatment was indirectly associated with life satisfaction (*β* = −0.42, *p* < 0.001) via resilience. Results of the bias-corrected method showed that the 95% CI of indirect effects was [−0.176, −0.104] and the 95% CI of the direct effect was [−0.543, −0.355], which suggests that resilience partially mediated the relationships between childhood maltreatment and life satisfaction among the Chinese student preschool teachers.

**Figure 2:**
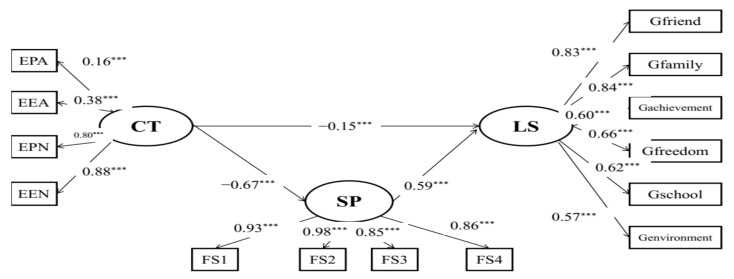
Mediation model of child maltreatment, life satisfaction, and social support. EPA: physical abuse; EEA: emotional abuse; EPN: physical neglect; EEN: emotional neglect; FS1: social support 1; FS2: social support 2; FS3: social support 3; FS4: social support 4; CT: childhood maltreatment; LS: life satisfaction; RE: resilience; SP: social support; *** *p* < 0.001.

Moreover, structural model B (Figure 2) with acceptable model fits (RMSEA = 0.081, CFI = 0.954, TLI = 0.938) indicated the mediating effects of social support on the relationships between childhood maltreatment and life satisfaction. Childhood maltreatment was indirectly associated with life satisfaction (*β* = −0.15, *p* < 0.001) via social support. The results of the bias-corrected method showed that the 95% CI of indirect effects was [−0.486, −0.341], and the 95% CI of the direct effect was [−0.281, −0.062], which suggests that social support partially mediated the relationship between childhood trauma and life satisfaction in the sample of Chinese student preschool teachers.

**Figure 3:**
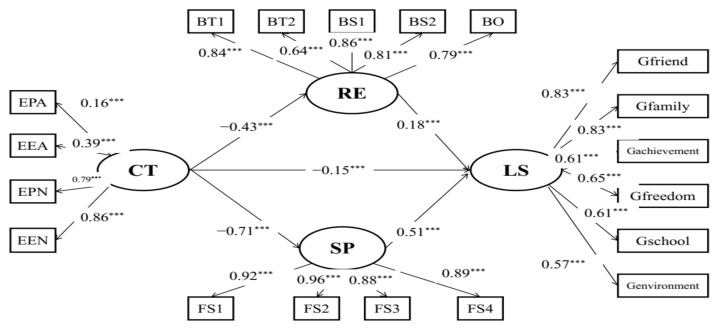
Mediation model of child maltreatment, life satisfaction, resilience, and social support. EPA: physical abuse; EEA: emotional abuse; EPN: physical neglect; EEN: emotional neglect; BT1: tenacity 1; BT2: tenacity 2; BS1: strength 1; BS2: strength 2; BO: optimism; FS1: social support 1; FS2: social support 2; FS3: social support 3; FS4: social support 4; CT: childhood maltreatment; LS: life satisfaction; RE: resilience; SP: social support; *** *p* < 0.001.

Additionally, structural model C (Figure 3) with acceptable model fits (RMSEA = 0.078, CFI = 0.937, TLI = 0.922) indicated the mediating effects of resilience and social support on the relationships between childhood maltreatment and life satisfaction. Childhood maltreatment was indirectly associated with life satisfaction (*β* = −0.15, *p* < 0.001) via resilience and social support. The results of the bias-corrected method showed that the 95% CI of indirect effects was [−0.530, −0.358], and the 95% CI of the direct effect was [−0.305, −0.063], which suggests that resilience and social support partially mediated the relationships between childhood trauma and life satisfaction in the sample of Chinese student preschool teachers.

### 3.4. Resilience and Social Support Mediated the Relationships between Subtypes of Childhood Maltreatment and Life Satisfaction

**Figure 4:**
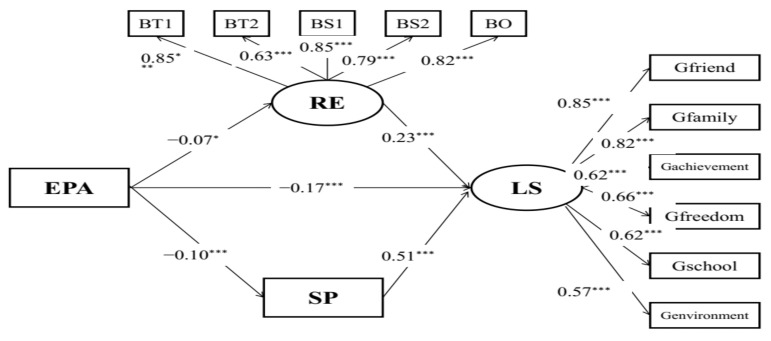
Mediation model of physical abuse, life satisfaction, resilience, and social support. EPA: physical abuse; BT1: tenacity 1; BT2: tenacity 2; BS1: strength 1; BS2: strength 2; BO: optimism; LS: life satisfaction; RE: resilience; SP: social support; * *p* < 0.05; *** *p* < 0.001.

The results showed that the structural model (Figure 4) with acceptable model fits (RMSEA = 0.07, CFI = 0.958, TLI = 0.940) indicated that resilience and social support partially mediated the relationships between physical abuse and life satisfaction (the 95% CI of the indirect effect was [−0.110, −0.018]; the 95% CI of the direct effect was [−0.292, −0.085]).

**Figure 5:**
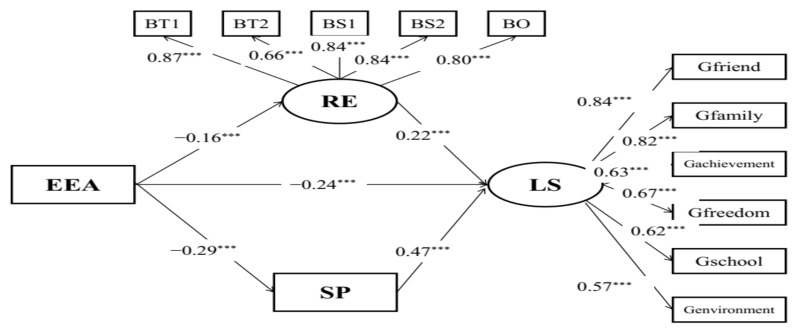
Mediation model of emotional abuse, life satisfaction, resilience, and social support. EEA: emotional abuse; BT1: tenacity 1; BT2: tenacity 2; BS1: strength 1; BS2: strength 2; BO: optimism; LS: life satisfaction; RE: resilience; SP: social support; *** *p* < 0.001.

The results showed that the structural model (Figure 5) with acceptable model fits (RMSEA = 0.07, CFI = 0.960, TLI = 0.943) indicated that resilience and social support partially mediated the relationships between emotional abuse and life satisfaction (the 95% CI of the indirect effect was [−0.215, −0.128]; the 95% CI of the direct effect was [−0.319, −0.173]).

**Figure 6:**
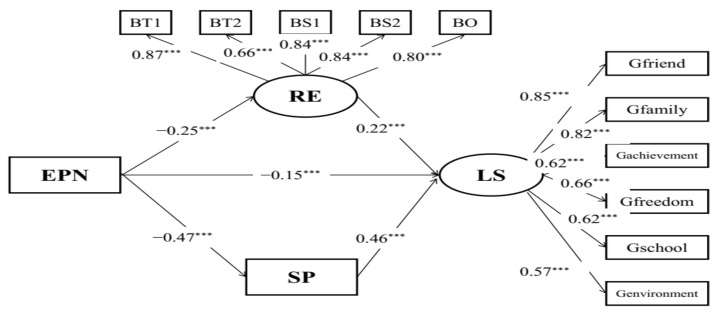
Mediation model of physical neglect, life satisfaction, resilience, and social support. EPN: physical neglect; BT1: tenacity 1; BT2: tenacity 2; BS1: strength 1; BS2: strength 2; BO: optimism; LS: life satisfaction; RE: resilience; SP: social support; *** *p* < 0.001.

The results showed that the structural model (Figure 6) with acceptable model fits (RMSEA = 0.07, CFI = 0.954, TLI = 0.935) indicated that resilience and social support partially mediated the relationships between physical neglect and life satisfaction (the 95% CI of the indirect effect was [−0.321, −0.225]; the 95% CI of the direct effect was [−0.233, −0.080]).

**Figure 7:**
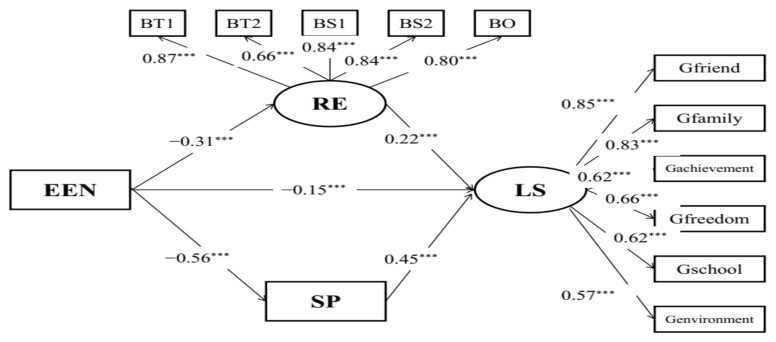
Mediation model of emotional neglect, life satisfaction, resilience, and social support. EEN: emotional neglect; BT1: tenacity 1; BT2: tenacity 2; BS1: strength 1; BS2: strength 2; BO: optimism; LS: life satisfaction; RE: resilience; SP: social support; *** *p* < 0.001.

The results showed that the structural model (Figure 7) with acceptable model fits (RMSEA = 0.07, CFI = 0.953, TLI = 0.933) indicated that resilience and social support partially mediated the relationships between emotional neglect and life satisfaction (the 95% CI of the indirect effect was [−0.371, −0.275]; the 95% CI of the direct effect was [−0.220, −0.080]).

## 4. Discussion

The current study verified the relationships between childhood maltreatment and life satisfaction among Chinese student preschool teachers and examined the mediating effects of resilience and social support in those relationships. To our best knowledge, this is the first study exploring the relationships and pathways between childhood maltreatment and life satisfaction in Chinese student preschool teachers. The results showed that childhood maltreatment, including physical and emotional maltreatment, was negatively associated with later life satisfaction, and resilience and social support mediated those relationships. These findings confirmed the assumptions based on ecological system theory that external environmental factors affect individuals’ emotions, behaviors, and attributions, which may provide evidence and suggestions for cultivating preschool teachers.

The results of the current study are consistent with previous studies, indicating that childhood maltreatment is negatively associated with resilience [26] and social support [18], as well as life satisfaction [12]. Specifically, physical and emotional maltreatment were negatively associated with later life satisfaction, social support, and resilience, which is consistent with previous studies [55,56,57]. Individuals who have experienced childhood maltreatment, including physical and emotional maltreatment, may have biased internal working models of interpersonal relationships [58], which may contribute to low levels of resilience and social support. Similarly, childhood maltreatment, as one adverse childhood experience, may have negative effects on individuals’ attributions [59], which may lead to low levels of life satisfaction. These findings suggest that childhood maltreatment, including physical and emotional maltreatment, may influence perceptions of daily life events and, in turn, may impair life satisfaction.

Moreover, the current study also found that resilience partially mediated the relationships between childhood maltreatment and life satisfaction, which is consistent with previous studies [47,60]. Further, resilience mediated the relationships between physical and emotional maltreatment and life satisfaction, respectively, which is consistent with previous studies which stated that resilience mediates the relationships between psychical and emotional maltreatment and later development [61,62]. The results suggest that childhood maltreatment not only has direct effects on life satisfaction, but also has indirect effects on life satisfaction via resilience. Childhood maltreatment, as a kind of negative interaction between a child and parent(s), may influence perceptions of self and others, which may contribute to low levels of self-esteem [63] and hostile attributions of others [64] and, in turn, lead to low levels of resilience. Individuals with high levels of resilience may have much more positive cognitions, which may decrease the effects of childhood maltreatment on life satisfaction. These findings suggest that individuals with high levels of resilience may have more interactions or positive feedback from others, which may give them much more bonds with others through which they can overcome adversity and improve their life satisfaction in adulthood.

Meanwhile, social support as a mediator in the relationships between childhood maltreatment and life satisfaction was confirmed in the current study, which is consistent with previous studies [65]. Similarly, social support also mediated the relationships between physical and emotional maltreatment and life satisfaction, respectively, which is consistent with previous studies which stated that social support mediated the relationships between physical and emotional maltreatment and later development [66,67]. Childhood maltreatment may influence perceptions of interpersonal relationships [68], which may contribute to low levels of social support, and these low levels of social support may lead to low levels of life satisfaction. However, high levels of social support may change the pathways from childhood maltreatment to life satisfaction. Individuals with high levels of social support may have solid networks with others, and they can share their feelings with friends, which may give them strength to overcome adverse experiences and enhance levels of life satisfaction. These findings suggest that individuals with high levels of social support may have positive interpersonal relationships, which may help them to bounce back from adversity.

Although the current study broadens the studies of teacher education and contributes to the literature to improve the quality of student preschool teachers, the current study also has some limitations. First, few male student preschool teachers were recruited in this study, so it might not present the whole picture of student preschool teachers. Future studies should contain many more male student preschool teachers and examine the gender differences in those relationships. Second, self-reported questionnaires were used in this study, which might have over-reported the situations of childhood maltreatment. Future studies should collect data from different sources, such as caregivers’ reports, which may make sure of the accuracy of information. Third, although the Life Satisfaction Scales Applicable to Chinese Adolescent Students has acceptable reliability and validity in Chinese college students, it was developed to assess life satisfaction in Chinese adolescent students, which may influence the results of life satisfaction. Future studies should apply scales for adults to assess college students’ life satisfaction.

Keeping the limitations in mind, some implications come from the current study. First, the current study confirmed that childhood maltreatment, including different subtypes, impairs life satisfaction in student preschool teachers, which suggests that childhood maltreatment might have a long-term effect on later development. Governments and communities should implement programs to raise awareness of protecting children and reduce occurrence of childhood maltreatment. For example, governments and communities may provide free lectures to popularize the outcomes of childhood maltreatment and to help parents know much more positive parenting strategies. Moreover, governments may create a system which registers the victims of childhood maltreatment and provides specific supports for them. Second, the current study verified the mediational roles of resilience and social support in the relationships between childhood maltreatment and life satisfaction, which suggests that high levels of resilience and social support might help individuals who have experienced childhood maltreatment overcome the negative effects. Governments and schools may provide services, such as psychological consulting about communicational skills, to help survivors of childhood maltreatment rebuild their social network, which may increase the levels of social support. Meanwhile, schools may provide some courses, such as self-management and positive psychology courses, to help student preschool teachers who have experienced childhood maltreatment reshape their internal working modeling surrounding the self and others and, in turn, increase their levels of resilience. Third, the results of the current study also suggest that adverse childhood experiences (e.g., childhood maltreatment) influence student preschool teachers’ life satisfaction, which may influence their work performance in turn. Governments and schools should pay much more attention to student preschool teachers who have experienced childhood maltreatment and provide supports for them. For example, schools may provide some courses that help survivors of childhood maltreatment learn communication skills and, finally, reach adaptation.

## 5. Conclusions

This study examined the relationships between childhood maltreatment and life satisfaction in Chinese student preschool teachers and explored the roles of resilience and social support in those relationships. In line with our predictions, childhood maltreatment, including physical and emotional maltreatment, was negatively associated with life satisfaction, and resilience and social support mediated those relationships. The findings suggest that childhood maltreatment not only has a direct relationship with life satisfaction, but also has an indirect relationship with life satisfaction via resilience and social support. Childhood maltreatment should be considered when selecting preschool teachers, and increasing the levels of resilience and social support should be attempted when cultivating student preschool teachers who have experienced childhood maltreatment.

## Figures and Tables

**Table 1:** The demographic information for participants (*n* = 1218).

		M(SD)/N (%)
Age		19.19 (0.94)
Gender	Girl	1162 (95.4)
	Boy	56 (4.6)
Only child	Yes	605 (49.7)
	No	613 (50.3)
Monthly family income	RMB 1000 and below	39 (3.2)
	RMB 1001 RMB~RMB 3000	182 (14.9)
	RMB 3001 RMB~RMB 5000	289 (23.7)
	RMB 5001 RMB~RMB 10,000	445(36.5)
	RMB 10,001 RMB~RMB 30,000	232 (19.0)
	RMB 30,001 and above	31 (2.5)

*Note*. M = mean age; SD = standard deviation; N = number; RMB = Chinese currency, RMB 7 ≈ USD 1.

**Table 2:** Means, standard deviations, and correlations between study variables.

Variables	*M*	*SD*	1	2	3	4
1 CT	1.44	0.67	1			
2 LS	5.37	1.13	−0.29 **	1		
3 RE	3.72	0.68	−0.11 **	0.36 **	1	
4 SP	5.67	1.15	−0.31 **	0.49 **	0.44 **	1

*Note*. CT: childhood maltreatment; LS: life satisfaction; RE: resilience; SP: social support. ** *p* < 0.01.

## Data Availability

The data will be valuable for request.
